# Cell type-stratified immunogenetic framework reveals immune drivers and therapeutic targets in kidney stone disease

**DOI:** 10.1016/j.omtn.2026.102964

**Published:** 2026-05-25

**Authors:** Dianjie Zeng, Lingfei Zhu, Jiachen Liu, Xiaorui Qiu, Leyi Chen, Chenming Wei, Yinhuai Wang, Zebin Deng

**Affiliations:** 1 Department of Urology, The Second Xiangya Hospital at Central South University, Changsha, Hunan 410011, China; 2 Department of Nephrology, The Second Xiangya Hospital at Central South University, Changsha, Hunan, China; 3 National Clinical Research Center for Metabolic Disease, Key Laboratory of Diabetes Immunology (Central South University), Ministry of Education, Changsha, China; 4 Department of Orthopedic, Xiangya Hospital at Central South University, Changsha, Hunan, China; 5 School of Medicine, Washington University, St. Louis, MO, USA

**Keywords:** MT: Clinical Applications, kidney stone disease, immune cell–specific regulation, single-cell eQTL, genetic regulatory architecture, colocalization, target prioritization

## Abstract

Despite growing recognition of immune involvement in kidney stone disease (KSD), its immunogenetic basis remains unclear. We applied a cell type-stratified immunogenetic framework, integrating single-cell *cis*-expression quantitative trait loci (*cis*-eQTL) data from the OneK1K immune atlas, to identify gene-cell type associations across four anatomical KSD subtypes. This approach revealed 80 colocalized gene-cell type pairs with strong genetic support. Natural killer (NK) cells and CD4^+^ naive/central memory T cells emerged as major contributors to disease risk, with consistent associations observed for *BCR*-NK cells and *RGS14*-CD4^+^/CD8^+^ T cells across all KSD subtypes. Functional and network analyses highlighted roles for MHC class II antigen presentation and T cell activation, implicating adaptive immunity in pathogenesis. Prioritized genes showed pleiotropic links to renal function, inflammation, and mineral metabolism—for example, *RGS14* was associated with improved eGFR and reduced urinary citrate; *GPX1* with impaired renal function and elevated CRP; *HCG25* and *DGKD* with higher calcium and uric acid. Clinical complication mapping further implicated these targets in osteoporosis, hypertension, and systemic inflammation. A multi-domain prioritization identified seven top targets—*RGS14*, *BCR*, *PRR3*, *HIST1H2BG*, *SLC44A4*, *POU5F1*, and *CTSS*—as clinically actionable. These insights may inform future strategies for risk stratification, early detection, and immune-targeted intervention in KSD.

## Graphical abstract

**Figure:**
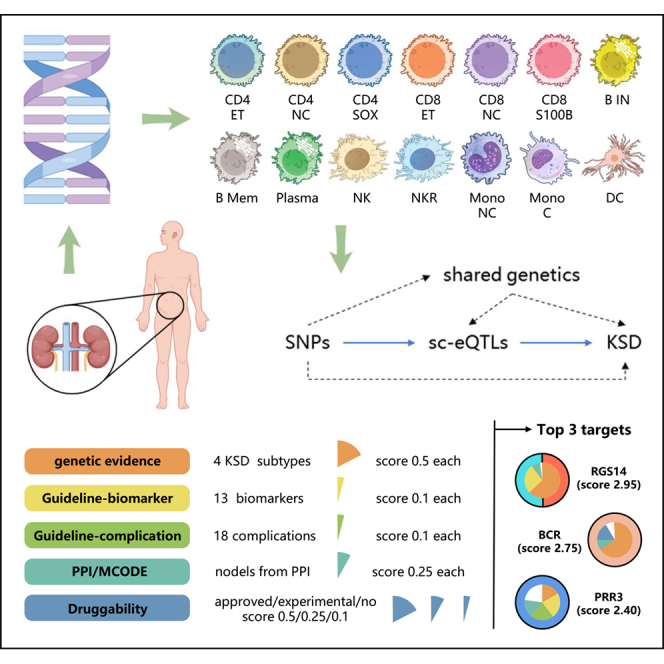

## Teaser

This study advances the understanding of kidney stone disease by mapping immune involvement to specific circulating immune-cell regulatory programs. The findings prioritize candidate genes and cell contexts that link genetic susceptibility with renal function, mineral metabolism, inflammation, complications, and therapeutic tractability.

## Introduction

Kidney stone disease (KSD), also known as urolithiasis, is a condition characterized by the precipitation of urinary solutes into crystalline aggregates within the urinary tract.^1^ Contemporary burden analyses highlight the persistently high—and in many regions, rising—incidence of KSD, leading to diminished quality of life and substantial healthcare expenditures.^2^^,^^3^ Clinically, KSD behaves as a chronic relapsing disorder: following an initial event, recurrence can occur in a large fraction of patients within a few years and continues to accumulate over time, establishing a prolonged at-risk trajectory.^4^^,^^5^^,^^6^ While endourological advances (e.g., ureteroscopy, shockwave lithotripsy) have improved stone clearance,^7^ procedures primarily address existing calculi rather than upstream biological programs that predispose to crystal deposition and recurrent disease,^8^ highlighting the need for mechanism-informed prevention and therapeutically tractable targets.

Historically, KSD pathogenesis has been framed primarily as a physicochemical process driven by urinary supersaturation, crystallization, and crystal retention on renal papillary surfaces.^9^^,^^10^^,^^11^ However, an expanding body of work supports a complementary view: stone formation may also be conceptualized as a tissue- and immune-mediated crystallopathy, in which crystals act as sterile danger signals that trigger inflammatory injury.^12^^,^^13^^,^^14^ Calcium oxalate (CaOx) crystals can activate innate immune sensing pathways and elicit IL-1β-dependent inflammation through the NLRP3-ASC-caspase-1 inflammasome in intrarenal mononuclear phagocytes.^15^^,^^16^^,^^17^ This provides a mechanistic bridge from mere crystalluria to sterile inflammation and tissue damage. Such inflammatory amplification may promote epithelial injury, cellular debris accumulation, and microenvironmental remodeling that favor crystal anchoring and growth.^18^^,^^19^^,^^20^

Immune cells are increasingly recognized as active effectors in crystal-associated disorders. In particular, neutrophil activation and neutrophil extracellular traps (NETs) formation can propagate sterile necroinflammation and matrix injury, with qualitative variation depending on crystal type, burden, and anatomical context.^21^ In kidney injury settings characterized by oxalate/CaOx deposition, coordinated macrophage–neutrophil responses—including NET formation—have been implicated in amplifying injury within the renal microenvironment.^22^^,^^23^^,^^24^ Translational observations in stone formers likewise report increased NET-related markers alongside renal calcium deposition and tubular injury signatures, supporting a plausible link between systemic/renal innate immune activation states and lithogenic susceptibility.^25^^,^^26^^,^^27^ Together with macrophage-mediated crystal sensing and clearance pathways, these data motivate a reframing of KSD as an immunometabolic disorder, where the composition and activation states of circulating and tissue immune subsets likely influence the initiation, propagation, and recurrence of stone disease.

**Figure 1:**
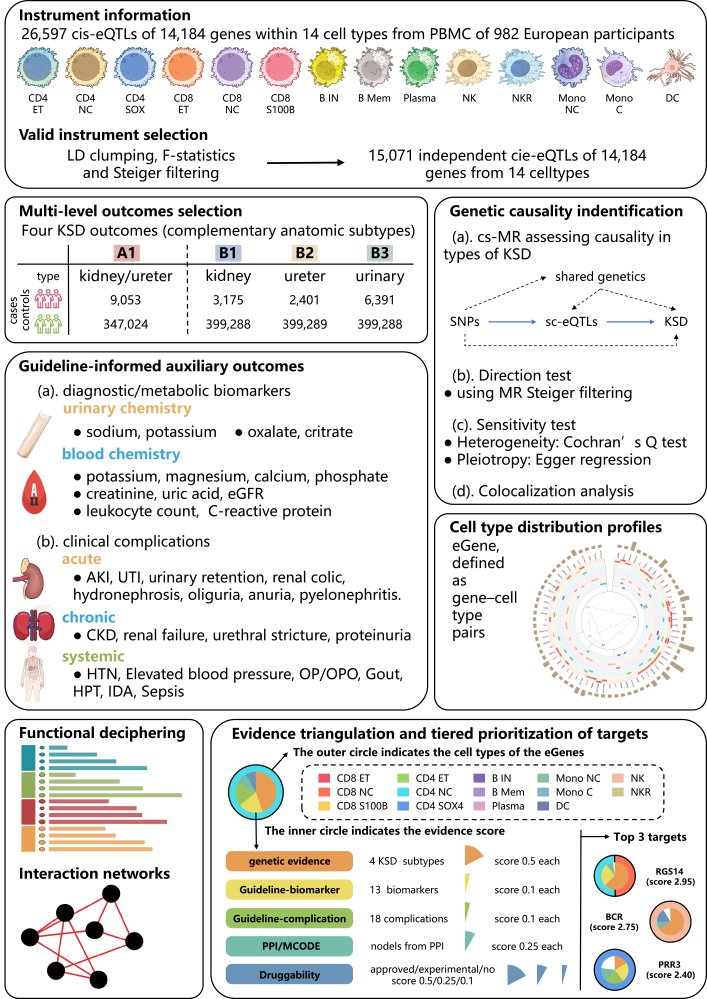
Overview of study design and analytical workflow Immune cell-specific gene expression data were obtained from single-cell *cis*-eQTLs in the OneK1K cohort. Summary statistics for kidney stone disease (KSD) were derived from genome-wide association studies (GWASs) conducted by the FinnGen Consortium and the UK Biobank.

Human genetics offers complementary leverage to connect these immune hypotheses to disease risk at scale. Recent genome-wide association studies (GWASs) and integrative post-GWAS analyses have mapped susceptibility loci and prioritized candidate genes, tissues, and druggable targets relevant to renal physiology.^28^^,^^29^ However, these associations seldom resolve the effector genes or, critically, the immune cell types and activation states through which risk is mediated. This is an especially important limitation when immune mechanisms act within specific leukocyte subsets rather than bulk cell populations. Closing this “variant → gene → cell type/state” gap is therefore essential for translating KSD genetics into interpretable mechanisms and actionable targets. This resolution is now feasible through emerging immune single-cell regulatory atlases and integrative frameworks. For instance, the OneK1K project combines single-cell RNA sequencing (scRNA-seq) with genotyping, mapping *cis*-expression quantitative trait loci (*cis*-eQTLs) across 14 peripheral immune cell types.^30^ Building on this, we integrated large-scale KSD GWAS with immune-cell regulatory genetics to pinpoint the immune subsets and regulatory programs most plausibly upstream of nephrolithiasis, thereby linking inherited susceptibility to crystal-induced sterile inflammation and providing a rational foundation for biomarker development and prevention-oriented intervention. The overall study design and analytical workflow are summarized in Figure 1.

## Results

### Summary of instrument selection for immune cell-specific gene expressions

We derived genetic instruments for immune cell–specific gene expression from single-cell *cis*-eQTL summary statistics spanning 14 circulating immune cell types in the OneK1K cohort (European ancestry; *n* = 982). In total, 26,597 conditionally independent *cis*-acting eQTLs were extracted as candidate instruments, capturing immune-cell-resolved regulatory effects across 14 immune cell types. In this study, the expression of a gene within a given immune cell type was treated as a distinct immune cell-specific gene expression profile (eGene).

**Figure 2:**
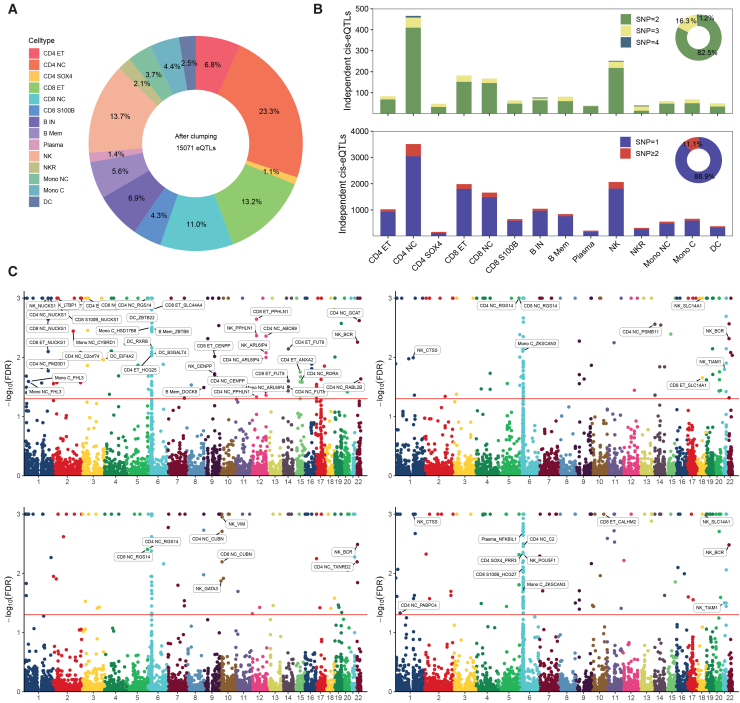
Selection of immune cell-specific genetic instruments (A) Distribution of independent *cis*-eQTLs across 14 immune cell types following linkage disequilibrium (LD) clumping. Cell-type abbreviations: CD4 NC, CD4^+^ naive/central memory T cells; CD8 NC, CD8^+^ naive/central memory T cells; CD4 ET, CD4^+^ effector memory/central memory T cells; CD8 ET, CD8^+^ effector memory/central memory T cells; CD4 SOX4, CD4^+^ T cells expressing SOX4; CD8 S100B, CD8^+^ T cells expressing S100B; NK, natural killer cells; NKR, NK recruiting cells; B IN, (immature B cells; B Mem, memory B cells; Mono NC, nonclassical monocytes; Mono C, classical monocytes; DC, dendritic cells; Plasma, plasma cells. (B) Bar plot showing the number of valid instruments per cell type. (C) Manhattan plot of cell type-stratified Mendelian randomization (cs-MR) results, highlighting significantly colocalized eGenes.

To obtain high-quality instruments for downstream analyses, we implemented a three-step selection protocol. First, we removed highly correlated variants by linkage disequilibrium (LD) clumping using the European panel of the 1000 Genomes Project as the reference, applying an LD threshold of r^2^ < 0.001 to retain representative and approximately independent *cis*-eQTLs. Second, we quantified instrument strength using F-statistics and excluded weak instruments. Third, we applied Steiger filtering to assess directionality by comparing the variance explained in the exposure versus the outcome and removing instruments more consistent with the reverse direction (outcome → exposure). In summary, 15,071 independent *cis*-eQTLs corresponding to 14,184 immune cell-specific eGenes across 14 immune cell types were retained as the final instrument set for subsequent analyses. Notably, the distribution of instruments and eGenes across immune cell types was stable before and after instrument selection, with consistent enrichment in CD4 naive/central memory T cells (CD4 NC) and natural killer cells (NK), whereas CD4 SOX4 and plasma cells contributed the fewest instruments and eGenes (Figures 2A and 2B; Table S1).

### Identification of putative candidate immune cell-specific signals for KSD

We next assessed the putative effects of immune cell-specific gene expression on KSD using a cell type-stratified framework across four independent GWAS outcomes: A1 (FinnGen, Calculus of kidney and ureter) and three UK Biobank phenotypes—B1 (Calculus of kidney, 594.1), B2 (Calculus of ureter, 594.3), and B3 (Urinary calculus, 594). All analyses were based on predominantly European-ancestry summary statistics, with exposure instruments derived from the OneK1K immune single-cell *cis*-eQTL dataset spanning 14 immune cell types. After harmonizing exposure (immune cell-specific gene expression profiles) and outcome (KSD) summary statistics, we computed MR estimates for 12,986 (A1), 13,538 (B1), 13,538 (B2), and 13,538 (B3) gene-cell type-trait combinations. Applying Benjamini-Hochberg correction (false discovery rate (FDR) <0.05), we identified 274 (A1), 248 (B1), 173 (B2), and 259 (B3) statistically significant immune cell-specific associations (Figure 2C; Table S4). To evaluate the robustness of MR estimates, we performed sensitivity analyses using Cochran’s Q test and MR-Egger regression. Most associations showed no evidence of heterogeneity or directional pleiotropy, supporting the reliability of our findings (Tables S5 and S6).

**Figure 3:**
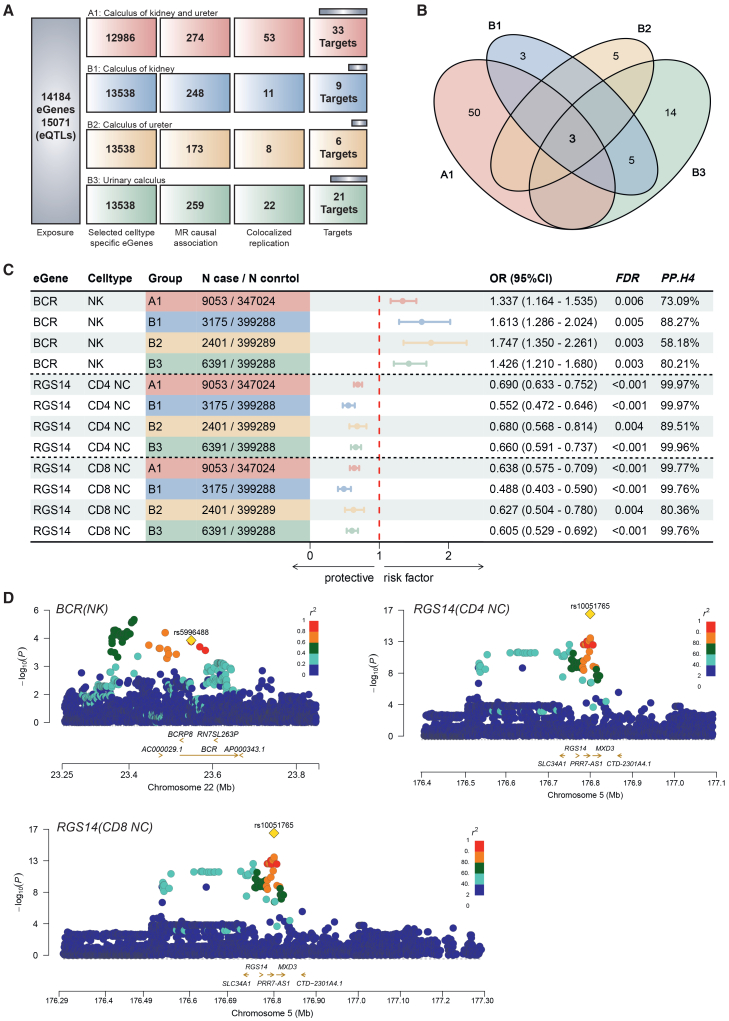
Summary of causal inference linking immune gene expression to KSD (A) Number of significant associations identified via cs-MR, sensitivity analyses, and Bayesian colocalization. (B) Venn diagram showing overlap of gene-outcome associations across the four KSD phenotypes. (C) Forest plot of eGenes with consistent effects across multiple KSD outcomes. (D) Regional association plots for *BCR* and *RGS14* loci, showing colocalized eQTL and GWAS signals for KSD.

To differentiate signals more consistent with shared genetic architecture from linkage disequilibrium, we performed Bayesian colocalization analysis, interpreting PP.H4 > 0.5 as moderate and PP.H4 > 0.8 as strong evidence for a shared genetic signal. Among the FDR-significant MR associations, 53 (A1), 11 (B1), 8 (B2), and 22 (B3) showed evidence of colocalization (Figures 3A and 3B). Altogether, we prioritized 80 immune gene-cell type pairs (MR associations with colocalization support) across the four KSD outcomes, implicating 58 unique genes with immune cell-specific effects across 14 immune subsets. Cross-outcome comparison revealed a subset of reproducible immune targets with consistent directions of effect. In total, eight eGenes demonstrated significant and directionally concordant associations across two or more KSD outcomes. Among them, three eGenes—breakpoint cluster region (*BCR*) in NK cells and *RGS14* in both CD4 NC and CD8 NC subsets—were supported across all four GWAS outcomes. An additional five eGenes showed consistent evidence in at least two outcomes (Figure 3C). Notably, all three pan-outcome eGenes also exhibited colocalization signals indicative of shared genetic signal in LD-based analysis (Figure 3D).

### Immune cell type-specific effects of gene expressions on KSD outcomes

**Figure 4:**
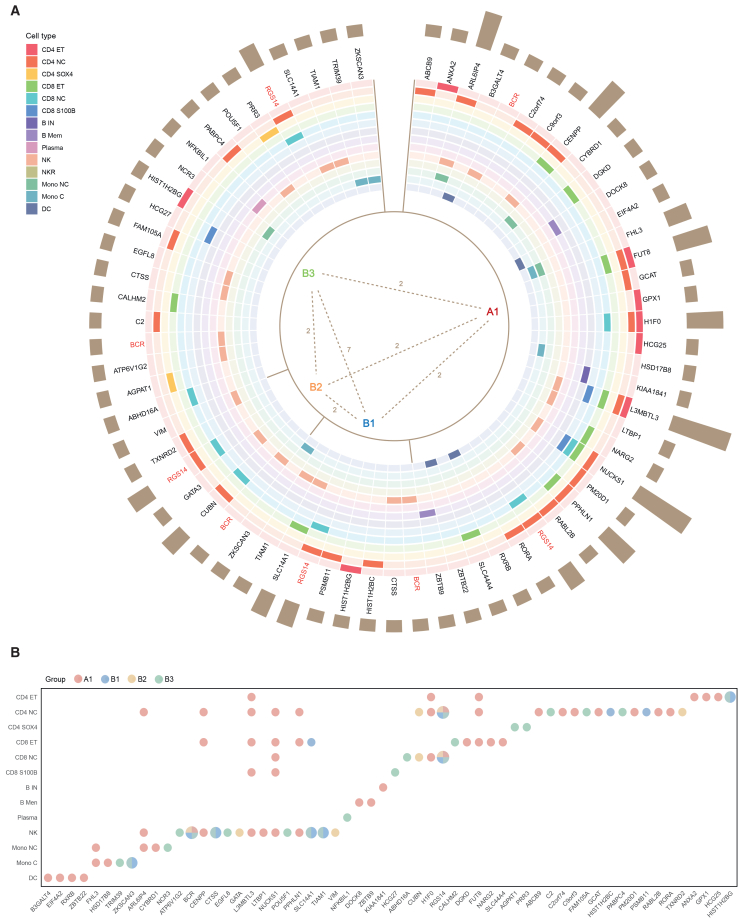
Immune cell-type specificity of top candidate gene-outcome pairs (A) Circular heatmap displaying the relationships between putative candidate genes, their associated immune cell types, and the four KSD outcomes. The outer ring shows a bar plot of the number of significant immune cell-type associations per gene. The middle ring displays gene labels annotated by immune cell type (color coded), and the inner sectors denotes outcome overlap. (B) Pie matrix summarizing outcome recurrence across cell types for high-frequency eGenes. The dot plot matrix shows immune cell types (rows) and genes (columns); dot size indicates the number of associated outcomes, and dot color reflects outcome identity.

We next evaluated the immune cell type-specificity of the genetically supported KSD targets. Among the 58 implicated genes, 42 showed evidence consistent with a cell type-restricted effect, with colocalized MR associations observed in only one immune cell type. In contrast, seven genes exhibited colocalized MR associations in three or more immune cell types, suggesting broader immune-mediated regulatory influences. For example, expression of *L3MBTL3* and *NUCKS1* in five immune cell types was associated with KSD risk (Figure 4A).

We further examined the subset of cross-outcome reproducible genes. Of the eight reproducible eGenes, four displayed effects in a single immune cell type, supporting marked cell type specificity even among the most robust candidates. At the cell-type level, supported associations appeared more frequent in CD4 NC and NK cells (Figure 4B). However, CD4 NC and NK cells also contributed the largest numbers of eGenes and instruments in the underlying sc-eQTL resource, indicating that apparent over-representation could be partially influenced by instrument availability.

### Guideline-informed auxiliary diagnostic biomarker mapping of prioritized immune targets

**Figure 5:**
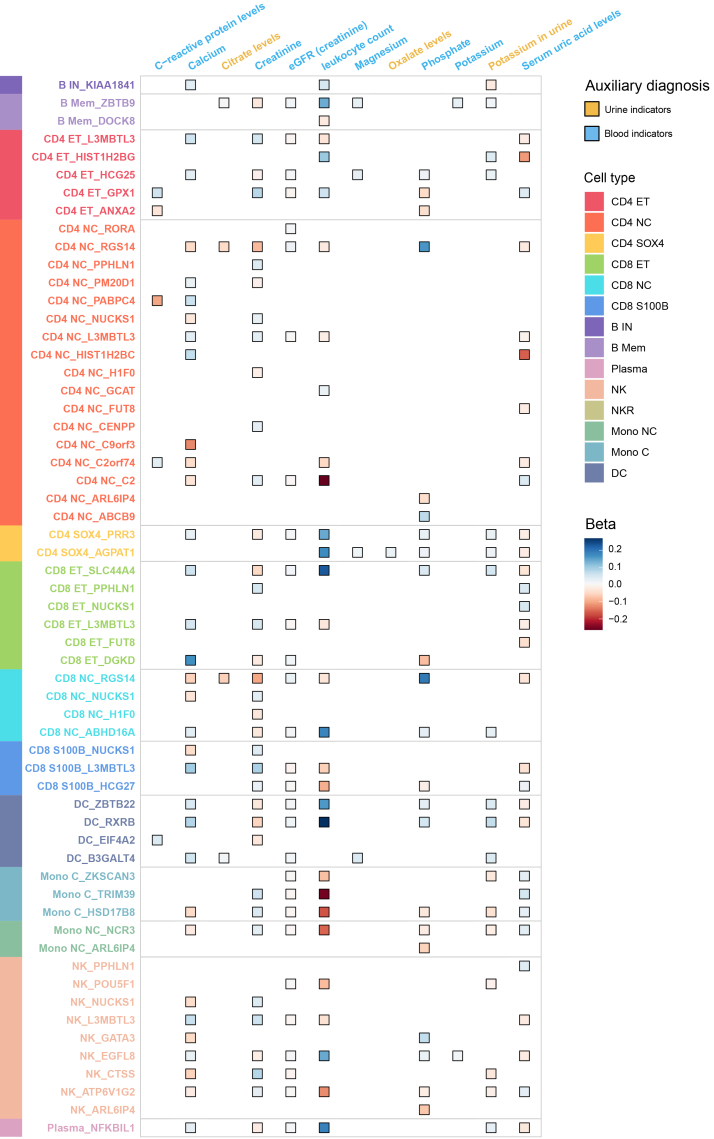
Biomarker associations of immune cell–specific eGenes Heatmap of eGene associations (rows) with clinically relevant biomarkers (columns), informed by clinical guidelines. Color scale indicates effect size (β). Side annotations distinguish biomarker type (e.g., blood or urine).

We mapped prioritized immune targets to guideline-recommended auxiliary diagnostic biomarkers for KSD, including urinary chemistry and blood-based measures of mineral metabolism, kidney function, and systemic inflammation. All associations reported below remained significant after multiple-testing correction; given heterogeneous biomarker biology, we assessed directional coherence within clinically interpretable modules and anchored interpretation to the primary KSD MR direction by classifying targets as risk-increasing or protective. Across biomarkers, we observed coherent signatures linking immune regulation to renal function, inflammation, and lithogenic urine chemistry. Notably, *GPX1* (risk-increasing in CD4 ET) mapped to a combined renal dysfunction-inflammation profile (creatinine↑, eGFR↓, inflammatory markers↑), whereas *RGS14* (protective across CD4 NC/CD8 NC) showed a reciprocal protective renal-function pattern (creatinine↓, eGFR↑) together with a citrate-lowering association. Because lower citrate is a recognized lithogenic feature, this biomarker pattern should not be interpreted as uniformly protective, but rather as evidence that *RGS14* may represent a pleiotropic regulatory signal connecting immune-cell gene regulation, renal function, and urinary chemistry. Mineral metabolism signals were frequent, exemplified by *HCG25* (risk-increasing) and *DGKD* (risk-increasing), both mapping strongly to serum calcium and related mineral-handling readouts. Collectively, these findings provide clinically interpretable context for genetically prioritized KSD immune targets within established diagnostic frameworks (Figure 5; Table S7).

### Guideline-informed complication mapping of prioritized immune targets

**Figure 6:**
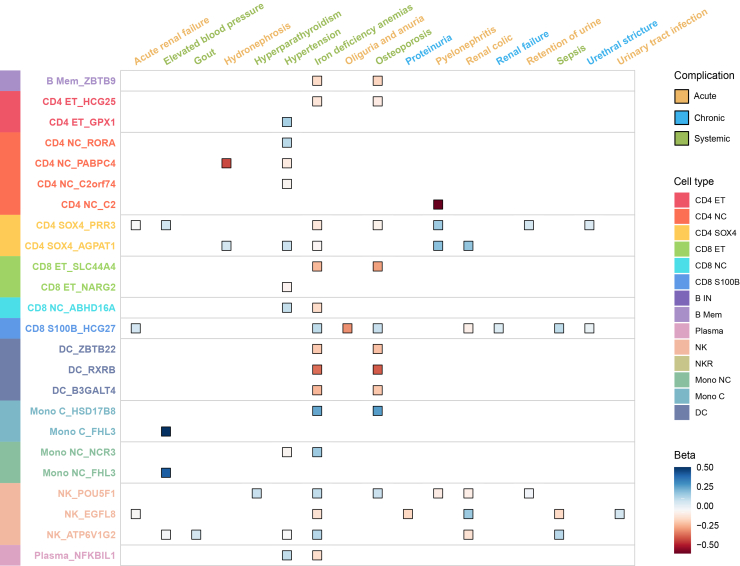
Mapping of immune targets to guideline-defined complications Heatmap showing associations between eGenes (rows) and KSD-related complications (columns), classified as acute, chronic, or systemic. Color intensity represents effect size (β). Column annotations indicate complication categories, enabling visualization of pleiotropic immune effects.

We next extended the guideline-informed phenome mapping to KSD-related complications spanning acute obstructive/infectious events, chronic renal sequelae, and systemic comorbidities. Acute complications clustered in specific immune contexts: in CD4 SOX4 T cells, *AGPAT1* and *PRR3* showed convergent links to acute presentations including pyelonephritis, renal colic, hydronephrosis, and urinary retention, consistent with a shared program relevant to symptomatic/obstructive disease. Infection-related endpoints also mapped to NK-cell programs (e.g., *EGFL8* with urinary tract infection and sepsis in opposite directions), highlighting heterogeneity in host-response pathways. Chronic renal sequelae were represented by signals in renal failure and proteinuria endpoints (e.g., *HCG27* in CD8 S100B; *EGFL8* in NK), while systemic comorbidities showed broad immune mapping, particularly for blood pressure traits. Elevated blood pressure signals were notable in monocyte lineages (*FHL3* in Mono C/Mono NC), supporting an immune–vascular interface in KSD comorbidity. Additional systemic signals included gout (*ATP6V1G2*) and hyperparathyroidism (*POU5F1*) in NK cells, and frequent mappings to osteoporosis and iron deficiency anemia across multiple immune contexts. Together, complication mapping suggests that genetically prioritized KSD immune targets extend beyond stone diagnosis into clinically recognizable acute, chronic, and systemic outcome domains, with both convergent clusters and directionally heterogeneous effects consistent with multiple pathophysiologic routes to morbidity (Figure 6; Table S8).

### Functional enrichment and interaction network analysis of genetically prioritized immune targets

**Figure 7:**
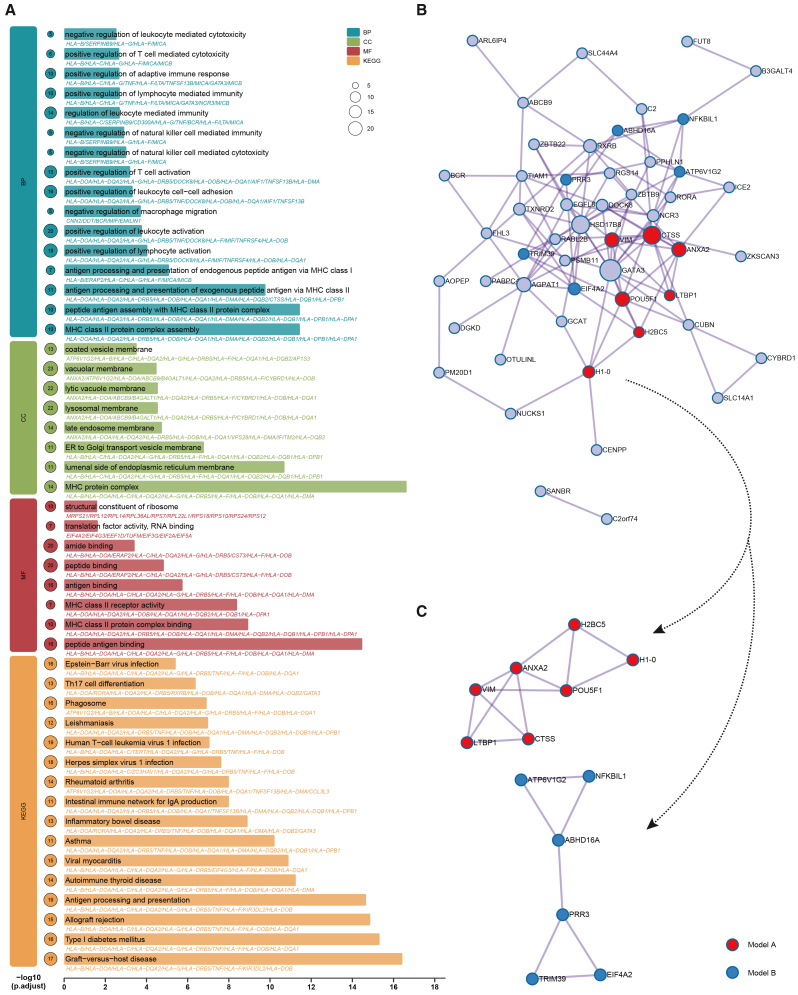
Functional enrichment and network context of prioritized targets (A) GO and KEGG enrichment of KSD-associated eGenes, showing significantly enriched immune and metabolic pathways. (B) Protein-protein interaction (PPI) network among prioritized immune genes. (C) Two representative gene modules identified via MCODE clustering, highlighting functional sub-networks.

To interrogate immune mechanisms enriched by the genetically prioritized KSD targets, we performed Gene Ontology (GO) (BP/CC/MF) and Kyoto Encyclopedia of Genes and Genomes (KEGG) analyses. Enrichment was dominated by antigen processing and presentation, particularly major histocompatibility complex (MHC) class II-related programs, together with broader lymphocyte/leukocyte activation, leukocyte adhesion, and T cell activation terms. GO cellular components localized these signals to the MHC complex and endomembrane trafficking compartments, while molecular functions highlighted peptide antigen binding and MHC class II activity/binding. KEGG results were concordant, prioritizing antigen processing and presentation and related HLA/T-cell-driven immune disease signatures (e.g., graft-versus-host disease, type 1 diabetes mellitus, allograft rejection) (Figure 7A). We then performed protein-protein interaction (PPI) network analysis for the genetically prioritized genes. Among the 58 unique genes included in this analysis, five of their encoded proteins showed little evidence of interaction with any other proteins, leaving 53 protein-coding genes for the PPI analysis (Figure 7B). A total of 13 of the 53 gene-coding proteins were further clustered into two modules using Metascape (models A and B) (Figure 7C).

### Druggability assessment and repurposing opportunities for prioritized immune targets

To evaluate translational potential of the genetically prioritized immune targets, we systematically cross-referenced drug-gene interactions and development-stage evidence using DrugBank, Open Targets, DGIdb, and ClinicalTrials.gov. This query identified 41 unique compounds mapped to 26 prioritized genes across multiple immune cell contexts. Of these, 33 compounds had a maximum development status of approved, spanning 21 genes, whereas ten compounds were experimental and mapped to five genes (*C2*, *C9orf3*, *H1F0*, *PSMB11*, and *TXNRD2*), highlighting a mixture of near-term repurposing candidates and longer-horizon opportunities (Table S9).

Several targets were linked to clinically established pharmacologic classes with clear tractability. For example, the reproducible NK-cell target *BCR* mapped to multiple approved tyrosine kinase inhibitors (dasatinib, imatinib, and ponatinib), providing an immediately actionable tractability signal. In addition, immune-processing genes within the antigen-presentation/endo-lysosomal theme were supported by approved or investigational compounds, including *CTSS* (approved fostamatinib; experimental petesicatib) and *ANXA2* (approved artenimol and fluocinolone acetonide; experimental lanoteplase). Metabolic or “systemic milieu”-linked targets also showed approved compound mappings, including *CUBN* (cyanocobalamin, hydroxocobalamin), *CYBRD1* (iron formulations), *RORA* (melatonin; citalopram), and *RXRB* (acitretin; adapalene). Notably, several mapped compounds were vitamins/supplements or broadly acting agents, underscoring that these annotations primarily inform target tractability and hypothesis generation rather than implying direct therapeutic efficacy for KSD.

### Evidence triangulation and tiered prioritization of KSD immune targets

**Figure 8:**
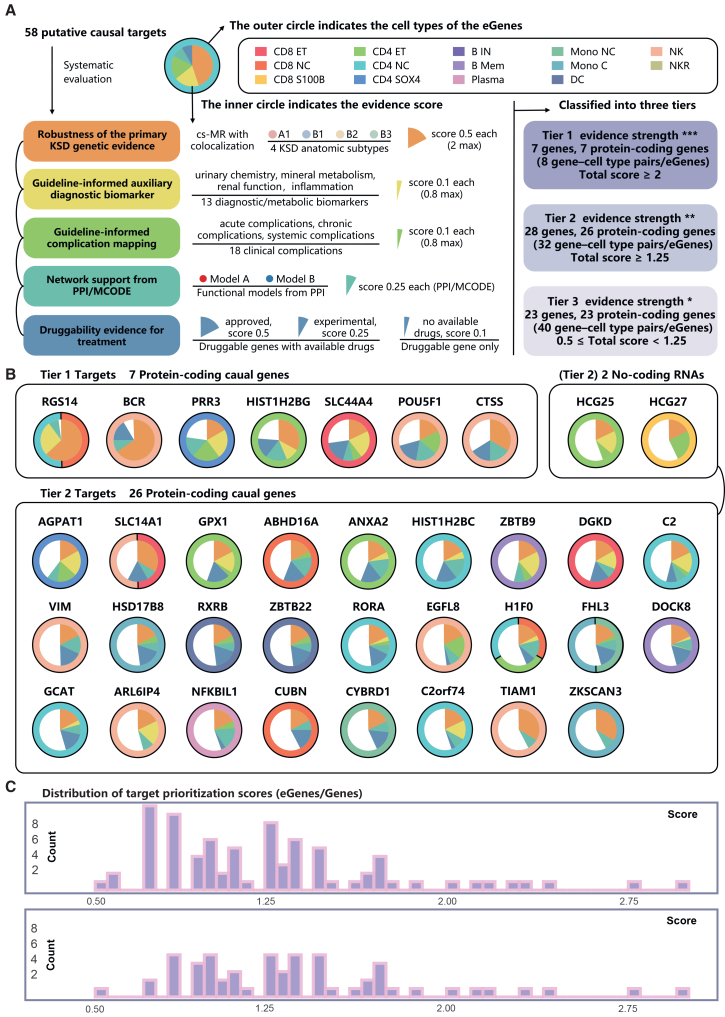
Prioritization and scoring of immune cell–specific KSD targets (A) Scoring framework for 80 eGene–cell type pairs, integrating five domains: GWAS replication, biomarker relevance, complication mapping, network context, and druggability. (B) Radial plot showing final prioritization of gene-level targets (only tier 1 and tier 2 shown). Outer ring indicates expression specificity across immune cell types; inner sectors represent score composition. (C) Histograms of prioritization scores: the upper image shows scores for all eGene-cell type pairs; the lower image aggregates gene-level scores (maximum per gene across cell types).

To prioritize genetically supported immune targets for downstream translational and mechanistic follow-up, we implemented a weighted evidence-triangulation framework integrating five domains: (1) robustness of the primary KSD genetic evidence across the four KSD GWAS outcomes, (2) guideline-informed auxiliary diagnostic biomarker mapping, (3) guideline-informed complication mapping, (4) network support from PPI/MCODE, and (5) druggability evidence. Scores were first assigned at the immune cell-specific target level (eGene, defined as gene-cell type pairs; *n* = 80), and gene-level prioritization was aggregated by selecting the highest-scoring eGene per gene, yielding a final ranked list of 58 unique genes (Figure 8; Table S10).

Using prespecified thresholds to define evidence tiers, seven genes were classified as tier 1 (max score ≥2.0), 28 genes as tier 2 (1.25 ≤ max score <2.0), and 23 genes as tier 3 (0.5 ≤ max score <1.25). The top-ranked candidates were dominated by targets with multi-cohort replication and/or strong translational support, led by *RGS14* (CD4 NC; score 2.95; also supported in CD8 NC) and *BCR* (NK; score 2.75), followed by a high-scoring, guideline-coherent target *PRR3* (CD4 SOX4; score 2.4). Additional tier 1 genes included *HIST1H2BG* (CD4 ET; score 2.3), *SLC44A4* (CD8 ET; score 2.15), *POU5F1* (NK; score 2.1), and *CTSS* (NK; score 2), reflecting convergence of immune network support and druggability evidence. Collectively, this triangulated ranking provides an interpretable shortlist of immune targets that integrate genetically supported associations, clinical guideline coherence, network context, and pharmacologic tractability.

## Discussion

The present integrative immune-genetic analysis provides genetic evidence supporting that KSD has a significant immunological component. The main contribution of this study is to refine this concept at immune-cell and gene-regulatory resolution. Importantly, the signals identified here are derived from circulating immune-cell *cis*-eQTLs from the OneK1K resource, rather than kidney-resident immune cells, renal epithelial cells, or stone-adjacent renal tissue. Through single-cell *cis*-eQTL Mendelian randomization and colocalization, we identified 58 genetically supported candidate genes linked to specific immune cell types. Some findings were robust across four independent GWAS outcomes (e.g., *BCR* in NK cells and *RGS14* in CD4 NC/CD8 NC). In addition, most of the implicated genes appear to act through one predominant immune cell subset, highlighting a striking cell-type specificity. This suggests that discrete arms of the immune system—rather than a generalized inflammatory state—drive different aspects of KSD risk. Indeed, only a few genes were reproducibly detected in more than one cell context, indicating that particular immune cells may have unique pathogenetic roles in stone formation. The colocalization evidence supporting these gene-trait links bolsters their interpretation and helps filter out false positives, yielding a prioritized set of immune-mediated targets for KSD. Finally, because our druggability analysis is annotation based, the therapeutic annotations should be interpreted as evidence of target tractability and hypothesis generation, rather than as evidence of therapeutic efficacy for KSD.

Notably, NK cells emerged with a significant enrichment of genetic signals in our analysis. Several candidate genes were identified predominantly via NK cell eQTLs, suggesting that innate immune surveillance and cytotoxic effector functions may influence stone formation.^31^ For example, the strongest colocalization signals (e.g., rs5996488 near *BCR*) were observed in NK cells, implicating genes involved in NK cell activation and cytokine release. This points to an innate immune axis in KSD where NK cells (or related innate lymphoid cells) might regulate renal inflammation or crystal clearance.^16^^,^^32^^,^^33^ Likewise, findings from a CD4^+^ T cell subset (denoted here as “CD4 NC” in our results) highlight the adaptive immune contribution.^34^ The CD4^+^ T cell compartment yielded a distinct set of eGenes—including some unique to this context—underscoring that T helper cell-mediated pathways are also at work. These cell-specific associations align with the functional enrichment we observed in antigen presentation and MHC class II-related pathways.^35^ Enriched GO terms indicate that many of the KSD-linked eGenes encode proteins involved in presenting antigens to T cells (e.g., HLA class II molecules, co-factors for antigen loading) or modulating T cell activation. This is biologically plausible: antigen-presenting cells (monocytes/macrophages, dendritic cells, and B cells) express MHC II to present peptides to CD4^+^ T cells, potentially including self-antigens released during crystal injury.^35^^,^^36^^,^^37^ The MHC II enrichment suggests that KSD risk variants may promote an exaggerated adaptive immune response—perhaps by enhancing the presentation of stone- or damage-related antigens to T helper cells, fueling local inflammation.

Integrating our genetic results with clinical phenotypes reveals that these immune genes do not act in isolation, but intersect with known biomarkers and complications of KSD. Several of the candidate eGenes map to pathways that influence diagnostic or metabolic markers of stone risk. For instance, one of the top replicated genes in our study, *RGS14*, has been independently associated with kidney stone susceptibility and implicated in regulation of calcium homeostasis. A functional variant in *RGS14* is thought to raise stone risk by altering calcium reabsorption or signaling, leading to elevated Ca^2+^ levels—a known lithogenic factor.^38^^,^^39^^,^^40^ The implication of *RGS14* in both our immune-focused analysis and in calcium metabolism suggests a nexus between immune gene variants and classical metabolic drivers of KSD. More broadly, many of our identified genes showed pleiotropic associations with systemic biomarkers. Some overlap loci influencing calcium-phosphate balance, vitamin D, or parathyroid hormone levels (e.g., *HCG25* and *DGKD*), whereas others intersect with pathways of uric acid metabolism and renal function (e.g., *GPX1*). This interplay implies that the genetic immune architecture of KSD partly acts by modulating systemic physiological traits that favor stone formation (such as hypercalciuria, hyperoxaluria, or low citrate levels),^41^^,^^42^ in addition to local immune responses. In essence, immune genes could contribute to a pro-lithogenic internal environment—for example, by subtle shifts in inflammatory cytokines that affect bone-mineral metabolism or kidney tubular handling of minerals.^43^^,^^44^^,^^45^

Crucially, our results also shed light on the well-documented complications and co-morbidities of kidney stones. KSD is not an isolated renal event; it carries increased risks of urinary tract infections,^46^ chronic kidney disease (CKD),^47^ and even progression to end-stage renal disease.^48^ The immune-linked genes we uncovered provide a biological framework for these connections. Many eGenes are known players in immune pathways that, when dysregulated, can cause systemic inflammation or autoimmunity.^49^ We observed that some KSD-risk genes are also associated with inflammatory or autoimmune conditions. This raises the possibility that a common genetic propensity underlies both kidney stones and other inflammatory disorders. In practical terms, an individual carrying such variants might have hyperactive immune responses that not only promote stone formation (e.g., via granuloma-like reactions to crystals) but also make them prone to autoimmune-mediated tissue damage or impaired infection control, thereby increasing CKD risk. It is intriguing to note that immune-related diseases were recently shown to increase KSD risk,^50^^,^^51^ and our genetic findings provide a potential explanation: the same immune perturbations that drive diseases like rheumatoid arthritis or psoriasis might also facilitate stone pathogenesis and its sequelae. Conversely, stones themselves elicit inflammation that could exacerbate kidney injury.^15^ Our identification of genes enriched in antigen presentation and T cell activation suggests that persistent immune activation in the kidney could link stone formation to fibrosis and loss of function.^13^ For example, if certain HLA class II variants heighten T cell responses to kidney self-antigens (perhaps unveiled by crystal-induced damage), this could promote chronic interstitial nephritis alongside stone formation.^49^ Such a mechanism would directly tie the immunogenetics of KSD to renal functional decline. In sum, by mapping KSD-associated immune genes to known clinical markers and complications, our study points to a broader immuno-metabolic syndrome, wherein immune dysfunction contributes to stone risk and to systemic outcomes like metabolic disturbances and kidney injury.^52^ This integrative perspective underscores that managing KSD might require addressing underlying immune and inflammatory factors, not just the crystals themselves.

Our findings have significant translational implications, as they highlight novel molecular targets and pathways for intervention in KSD. We prioritized 58 genes using a tiered system that considered replication across analyses, links to biomarkers and complications, membership in the PPI network, and druggability. This systematic ranking is intended to accelerate the translation of genetic insights into clinical solutions. At the top tier, we identified seven key candidate targets with multi-faceted evidence supporting their importance in KSD and potential tractability for therapy. Notably, *BCR* and *RGS14* emerged as exemplars of high-priority targets. *BCR* is of particular interest; beyond its famous role in the BCR-ABL oncogenic fusion, it encodes a Rho GTPase-activating protein that regulates leukocyte function.^53^ In macrophages, *BCR* (together with its homolog Abr) serves as a critical negative regulator of Rac1 signaling, thereby modulating processes like cell motility and phagocytosis.^54^ This is highly relevant to KSD pathogenesis—effective migration of macrophages to crystal sites and their ability to phagocytose crystals can determine whether nascent stones are cleared or allowed to grow.^55^ A genetic predisposition affecting *BCR* could tilt this balance. The fact that *BCR* sits in a known druggable pathway (Rho GTPase signaling) is encouraging; while *BCR* itself is not yet a direct drug target, the pathway has been pharmacologically manipulated in other contexts (e.g., Rho/Rac inhibitors).^56^

Likewise, *RGS14*—a scaffolding protein regulating G-protein signaling—connects immune cell signaling with renal mineral handling, and thus represents a node where immune modulation might influence stone-relevant physiology.^57^^,^^58^^,^^59^ Not only has *RGS14* been linked to calcium level regulation, but RGS proteins are generally considered druggable (several small molecules targeting RGS-GPCR interactions are in development).^60^ However, the *RGS14* signal should also be interpreted with caution. Although *RGS14* showed reproducible protective associations with the primary KSD outcomes in CD4 NC and CD8 NC cells, its auxiliary biomarker profile was directionally complex. In particular, the association with lower urinary citrate is not straightforward from a stone-risk perspective, because hypocitraturia is a recognized risk factor for kidney stone formation. This finding suggests that *RGS14* may act as a pleiotropic regulatory locus linking immune-cell gene regulation, renal function, and urinary chemistry, rather than through a simple uniformly protective pathway. Moreover, we cannot exclude the possibility that immune-cell-specific regulatory signals partly reflect secondary or bidirectional responses related to crystal deposition, epithelial injury, or inflammatory remodeling.

Finally, we acknowledge certain limitations of our study. First, our analysis was constrained by the available data sources and predominantly evaluated individuals of European ancestry. Genetic mechanisms can vary across populations, and it will be important to extend this work to diverse cohorts to ensure that the identified candidate genes are globally relevant. Second, while MR and colocalization provide strong evidence for genetic association, the findings are still statistical inferences. Experimental validation will be crucial—for example, using *in vitro* cell culture or *in vivo* animal models to confirm that modulating the expression of a top gene in the relevant immune cell alters crystal formation or renal injury. Such functional follow-up is non-trivial, especially given the complexity of immune-kidney interactions. Third, our single-cell eQTL data were derived from blood immune cells; these may not capture tissue-resident immune cells in the kidney that could have context-specific eQTL effects. It’s possible that additional immune genes might emerge if, say, renal macrophages or resident T cells were profiled. Despite these caveats, the convergence of multiple analytical lines in our study provides confidence that the signals we report are biologically meaningful.

In conclusion, this study provides a cell type-stratified immunogenetic prioritization framework for KSD. Rather than simply confirming that immunity is involved in stone disease, our analysis identifies specific circulating immune-cell contexts and candidate regulatory genes that may connect inherited susceptibility with renal function, mineral metabolism, inflammatory profiles, and clinically relevant complications. The prioritized targets included *RGS14*, *BCR*, *PRR3*, *HIST1H2BG*, *SLC44A4*, *POU5F1*, and *CTSS*. Future studies are warranted to understand the causal mechanisms, optimal targets, efficacy and safety of these targets, and their potential benefits on preventing and treating KSD.

## Materials and methods

### Study overview

We aimed to systematically prioritize immune cell-specific regulatory targets potentially upstream of KSD. To this end, we integrated single-cell *cis*-eQTL data across 14 peripheral immune cell types from the OneK1K resource with GWAS summary statistics for four KSD outcomes. We used a cell type-stratified Mendelian randomization (cs-MR) and colocalization framework to evaluate associations between genetically proxied immune-cell regulatory programs and KSD risk across cell types. To contextualize immune-linked targets in clinically relevant terms, we curated a panel of diagnostic, metabolic, and complication-related traits based on clinical guidelines, and tested prioritized genes against these outcomes using the same genetic pipeline. Functional interpretation included pathway enrichment, protein-protein interaction network analysis, and module detection. Finally, we assessed translational relevance by mapping prioritized targets to approved or investigational drugs using curated drug-gene databases. Based on these multi-layered criteria, we developed a scoring and tiering system to rank candidate targets by their therapeutic potential. A schematic of the overall pipeline is shown in Figure 1.

### Instrument selection and validation

Genetic instruments were derived from single-cell *cis*-eQTL summary statistics across 14 peripheral immune cell types from the OneK1K resource, generated in European-ancestry donors with population-scale peripheral blood mononuclear cell (PBMC) scRNA-seq and genotype data.^30^ We retained conditionally independent *cis*-eQTLs associated with immune cell-specific gene expression at *p* < 0.005 as candidate instruments.^61^ To ensure independence between variants, we performed linkage disequilibrium (LD) clumping using an r^2^ < 0.001 threshold with a European reference panel from the 1000 Genomes Project.^62^ Instrument strength was quantified using the F-statistic (computed from the SNP-expression association), and variants with F < 10 were excluded to reduce weak-instrument bias.^63^ Details on instrument strength metrics, exclusion rationale, and the number of instruments retained per immune cell type and gene are provided in Table S1.

### Outcome GWAS selection (primary KSD)

To ensure robustness across biobank settings and clinical definitions, we selected four genetically informed KSD outcomes representing complementary case ascertainment strategies and anatomic subtypes. All outcome GWAS summary statistics were derived from predominantly European-ancestry participants and were harmonized to a consistent genome build and allele orientation prior to downstream analyses.

A1 (from FinnGen, https://www.finngen.fi/en): the primary KSD outcome was obtained from the FinnGen consortium (Calculus of kidney and ureter, ICD-10 N20), leveraging linked nationwide registry-based diagnoses, which included 356,077 participants with 9,053 cases and 347,024 controls. B1–B3 (from UK Biobank, https://www.ukbiobank.ac.uk/): to capture phenotype heterogeneity and site-specific effects, we additionally analyzed three UK Biobank outcomes that separate kidney stones, ureteral stones, and broader urinary calculus diagnoses. B1: calculus of kidney (phenotype 594.1, included 3,175 cases and 399,288 controls). B2: calculus of ureter (phenotype 594.3, included 2,401 cases and 399,289 controls). B3: urinary calculus (phenotype 594, included 6,391 cases and 399,288 controls).

Across these outcomes, we retained autosomal variants and ensured consistent alignment between exposure and outcome datasets. Detailed phenotype definitions and cohort descriptions are summarized in Table S2. As all data were aggregated and fully anonymized, and ethical approvals had been obtained by the original GWAS consortia, no additional ethical review was required for this analysis.

### Single-cell *cis*-eQTL-based Mendelian randomization analysis

Genetic inference analyses were performed using the filtered and LD-pruned *cis*-eQTL instruments described previously, stratified by immune cell type. For each gene-cell type combination, the directionally anchored effect on KSD risk was estimated separately in each of the four KSD GWAS outcomes (A1, B1–B3). When a gene-cell type pair had a single eligible instrument, we applied the Wald ratio.^64^ When two or more instruments were available, we used a generalized inverse-variance weighted (gIVW) approach,^65^ which allows incorporation of residual correlation structure among instruments when applicable.

All exposure and outcome summary statistics were harmonized prior to analysis to ensure consistent effect allele alignment. MR analyses were implemented in R (e.g., using the TwoSampleMR framework for harmonization/estimation),^66^ and multiple comparisons were controlled using the Benjamini-Hochberg FDR, with FDR <0.05 considered statistically significant. Reporting and analytical choices were guided by STROBE-MR principles.^67^ Directionality was evaluated using Steiger filtering, which compares the variance explained in the exposure versus the outcome^68^; instruments more consistent with the reverse direction (outcome → exposure) were removed prior to estimation. Only variants passing all filtering steps were included in the final analyses.

To evaluate robustness and detect potential violations of core assumptions, we conducted sensitivity analyses where instrument number permitted. Heterogeneity across instruments was assessed using Cochran’s Q test for each gene-cell type-outcome combination (Q test *p* < 0.05 indicating heterogeneity).^69^ Horizontal pleiotropy was evaluated using MR-Egger regression, with the intercept term tested for deviation from zero; a non-zero intercept suggests unbalanced pleiotropy that could bias inference.^70^

### Bayesian genetic colocalization

To distinguish signals consistent with a shared genetic signal from associations potentially driven by LD, we performed Bayesian colocalization using the coloc R package.^71^ For each gene-cell type-KSD pair, we extracted *cis*-eQTL and GWAS summary statistics in a ±100 kb window around the gene boundary and estimated posterior probabilities for the standard colocalization hypotheses, focusing on the posterior probability for hypothesis 4, representing a shared genetic signal (PP.H4). Colocalization evidence was interpreted a priori as moderate when PP.H4 > 0.5 and high when PP.H4 > 0.8.^72^ For downstream analyses (including guideline-informed biomarker/complication outcomes and functional annotation), we prioritized robust immune targets supported by both (1) statistically significant genetic inference evidence (FDR <0.05) and (2) colocalization evidence (PP.H4 > 0.5, with PP.H4 > 0.8 denoting high-confidence pairs).

### Guideline-informed auxiliary outcomes: Diagnostic/metabolic biomarkers and clinical complications

To contextualize KSD-associated immune targets in clinically interpretable pathways and to test coherence with established evaluation frameworks, we curated a set of auxiliary diagnostic/metabolic traits and KSD-related complications based on major urolithiasis guidelines from the American Urological Association (AUA, https://www.auanet.org/) and the European Association of Urology (EAU, https://uroweb.org/).

Specifically, guideline recommendations emphasize metabolic evaluation using 24-h urine collections (including, at minimum, urinary sodium, potassium, oxalate, citrate, and related measures) as well as serum/clinical chemistry markers relevant to stone risk profiling and renal function. In parallel, both guidelines highlight that stone disease can present with, or lead to, clinically meaningful acute obstructive/infectious events and longer-term renal and systemic sequelae (e.g., hydronephrosis, urinary tract infection, acute kidney injury, and sepsis).

We therefore assembled follow-up outcomes in three prespecified categories: (1) urinary biomarkers, including sodium, potassium, oxalate (ethanedioate) levels, and citrate levels; (2) blood biomarkers, including potassium, magnesium, calcium, phosphate, creatinine, leukocyte count, serum uric acid levels, estimated glomerular filtration rate (creatinine-based), and C-reactive protein levels; and (3) clinical complications, grouped a priori into acute, chronic, and systemic sequelae, including acute complications (urinary retention, acute renal failure/acute kidney injury, urinary tract infection, renal colic, hydronephrosis, oliguria and anuria, and pyelonephritis), chronic complications (urethral stricture not specified as infectious, chronic renal failure/CKD, renal failure, and proteinuria), and systemic complications (hypertension, elevated blood pressure reading without diagnosis of hypertension, osteoporosis, gout, hyperparathyroidism, iron deficiency anemias, and sepsis).

Only immune cell targets supported by both (1) statistically significant evidence in the primary KSD genetic inference analyses and (2) colocalization evidence were carried forward to these auxiliary outcomes. For all auxiliary outcomes, we applied the same harmonization procedures, estimators, and sensitivity analyses as in the primary KSD analyses. Detailed phenotype definitions, GWAS sources, and sample sizes for all auxiliary outcomes are provided in Table S2.

### Functional enrichment and PPI network analysis

To characterize the biological functions of immune targets supported by both genetic inference and colocalization, we performed GO and KEGG enrichment analyses using Metascape (http://metascape.org/). Enrichment results were corrected for multiple testing; terms with adjusted *p* < 0.05 were considered significant. To further explore functional connectivity and identify potential hub targets, we constructed PPI networks, and densely connected subnetworks were detected using the Molecular Complex Detection (MCODE) algorithm to highlight putative functional modules and hub genes.^73^

### Drug target prioritization and therapeutic annotation

To assess translational potential, we systematically annotated prioritized immune targets for druggability and therapeutic development status by cross-referencing complementary drug/target resources. We used DrugBank (https://go.drugbank.com/) to identify approved and experimental drugs and curated drug-target relationships, Open Targets Platform (https://platform.opentargets.org/) to obtain integrated target-disease evidence and tractability annotations, and Drug-Gene Interaction Database (DGIdb, https://dgidb.org/) to capture curated drug–gene interaction records and druggability categories. We additionally queried ClinicalTrials.gov (https://clinicaltrials.gov/) to confirm whether a target (or a target-modulating intervention) had registered clinical studies and to distinguish clinical-stage targets from those supported only by preclinical or literature-based evidence.

Targets were classified into three prioritization categories based on the strongest available translational evidence: (1) targets with at least one approved therapy that directly acts on the target; (2) targets supported by clinical-stage development evidence (e.g., registered clinical studies and/or investigational compounds) but lacking an approved therapy; and (3) targets with evidence of druggability or tractability but without approved therapies or registered clinical studies. To operationalize category 3 as “druggable targets without clinical development,” druggable genes were primarily identified using the Drug=Gene Interaction Database (DGIdb). We downloaded the DGIdb Categories Data (February 2022 release), which annotates genes classified as druggable and maps them to Entrez Gene identifiers, and used this dataset as the reference druggable gene set (Table S3). As an external validation, we additionally referenced the druggable genome compendium described by Finan et al.^74^ Genes were assigned to category 3 if they were included in either the DGIdb or Finan druggable gene sets but showed no evidence of approved drugs (DrugBank and Open Targets Platform) and no registered clinical trials (ClinicalTrials.gov) at the time of querying. These genes were therefore interpreted as druggable targets lacking active clinical-stage development.

### Statistical analysis and reproducibility

All statistical analyses were conducted using R (v.4.2.3) and Python (v.3.8) unless otherwise specified. The primary MR results are reported with FDR adjusted *p* values. All code and processed results will be made available upon reasonable request.

## Data and code availability

All data generated or analyzed during this study are included in this article and its supplemental information files. The datasets used and/or analyzed during the current study are available from the corresponding author upon reasonable request.

## Acknowledgments

The authors thank all investigators and participants who contributed to the GWAS and transcript-level datasets used in this study. This work was made possible by the generous sharing of summary statistics from the OneK1K project, FinnGen Consortium, UK Biobank, and other public databases. This work was supported by the 10.13039/501100001809National Natural Science Foundation of China (82500843); the National Postdoctoral Researcher Program (GZC20251346); the China Postdoctoral Science Foundation–Hunan Province Joint Special Funding Program (2025T020HN); and the 10.13039/501100004735Natural Science Foundation of Hunan Province (2026JJ60067). The data analyzed in this study were derived from publicly available, de-identified summary-level datasets, and ethical approval and informed consent had been obtained in the original studies. Therefore, no additional ethical review was necessary for the present secondary analysis.

## Author contributions

D.Z. and J.L. conceptualized and designed the study. D.Z., J.L., and L.C. contributed to the study design and data acquisition. L.Z., J.L., and Y.W. contributed to data interpretation and manuscript revision. D.Z., L.Z., C.W., and Z.D. conducted the statistical analyses and drafted the manuscript. All authors reviewed and approved the final manuscript. Z.D. will serve as the corresponding author for future inquiries regarding this study.

## Declaration of interests

The authors declare no competing interests.
